# Role of vitamin C in preventing of COVID-19 infection, progression and severity

**DOI:** 10.3934/microbiol.2022010

**Published:** 2022-03-30

**Authors:** Umar Shahbaz, Nazira Fatima, Samra Basharat, Asma Bibi, Xiaobin Yu, Muhammad Iftikhar Hussain, Maryam Nasrullah

**Affiliations:** 1 Key Laboratory of Carbohydrate Chemistry & Biotechnology, Ministry of Education, School of Biotechnology, Jiangnan University, Wuxi, Jiangsu, China; 2 Key Laboratory of Industrial Biotechnology, Ministry of Education, School of Biotechnology, Jiangnan University, Wuxi, Jiangsu, China; 3 Laboratory Animal Center, Institute of Atherosclerotic Disease, Xi'an Jiaotong University, No. 76 Yanta West Road, Xi'an ,710061, Shaanxi Province, China; 4 School of biotechnology, Jiangnan university, Wuxi, China; 5 NIAB (Nuclear Institute for Agriculture & Biology), punjab Pakistan; 6 The Key Laboratory of Microbiology and Parasitology Anhui, School of Basic Medical Sciences; and Laboratory Diagnostics of the First Hospital of Anhui Medical University, Hefei 230032, China

**Keywords:** COVID-19, vitamin C, anti-oxidant, antiviral, SARS-CoV-2, sepsis

## Abstract

Vitamin C stands as an essential water-soluble vitamin, antioxidant and has been shown to enhance immunity. SARS-CoV-2 has been spreading rapidly across the worldwide, several cellular processes of innate and adaptive immunity are aided by vitamin C, which strengthens the immune system overall. Multiple lines of evidence in the literature associate vitamin C with antioxidant, anti-inflammatory, anticoagulant and immunomodulatory actions. Pneumonia and sepsis patients had poor ascorbic acid status and high oxidative stress, according to many studies. Pneumonia patients who get vitamin C may have less severe symptoms and a longer course of the illness if they do. To standardize plasma levels in sepsis patients, gram measurements of the vitamin must be administered intravenously (IV). This intervention has been shown in a few trials to reduce mortality. COVID-19 management in China and the United States has exhibited remarkable results when using a high percentage of intravenous vitamins C. It's acceptable to include vitamin C in the COVID-19 treatment protocol as a secondary measure based on the current active clinical studies looking at the impact of vitamin C on the management of COVID-19. Patients with hypovitaminosis C or severe respiratory illnesses, such as COVID-19, may benefit from taking vitamin C, due to its good safety profile, simplicity of use, and potential for rapid production scaling. The study's goal was to see whether high dosage intravenous vitamin C had any impact on individuals with severe COVID-19 (HDIVC). Finally we discuss recent research that has been published on the efficacy of vitamin C administration in the treatment of viral infection and life-threatening conditions. The purpose of this manuscript is to summarise existing research on the efficacy of vitamin C as a treatment for COVID-19 and to discuss possible explanations for why it may work in some individuals but not in others.

## 1. Introduction

The COVID-19 (SARS-CoV-2) virus was originally discovered in Wuhan, China. It was initially reported to the WHO on December 31, 2019, and is now spreading across the globe at an alarming rate. It is caused by infection with Severe Acute Respiratory Syndrome Coronavirus 2 (SARS-CoV-2). As of today, SARS-CoV-2 has infected millions of individuals globally, causing an asymptomatic illness, inadequate therapy, and lengthy incubation periods. There are more than 184 vaccinations in pre-clinical trials (at the time of writing), with 35 vaccines in phase 1, 34 in phase 2, 28 in phase 3, and 17 presently being given to the general public. To date, various vaccines (Pfizer/BioNTech, Moderna, Johnson & Johnson, AstraZeneca, Sputnik, Sinovac, Novavax, Sinopharm, Bharat Biotech, CanSino, etc.) have been approved by the UK, CA, IN, AR, EU, CN, AR, BR, and MX for emergency use. More than 54.9% of the world's population has been full vaccinated , with 10.5 billion doses given in over 67 countries [1]. Even in low-income nations, just 1.9 percent of people had obtained the single dose of the vaccine thus far. Even yet, there's a long way to go until we've vaccinated 70 percent of the global population and ended this epidemic. As a result, regulating COVID-19 necessitates aggressive therapy that includes large doses of micro-nutrients like vitamin C. Vitamin C is a water-soluble vitamin that has been shown to benefit individuals with severe conditions including cancer and HIV/AIDS [2]. An anti-oxidant, it reduces inflammation and keeps fluid in the endothelium by keeping cellular immunity strong [3]. Endogenous catecholamines are produced with its help, and it contributes as a cofactor in the mechanism. The biosynthetic cascade in humans and a few other species has been disrupted by the loss of crucial enzymes [4],[5]. Intravenous high-dose vitamin C therapy is currently being evaluated for efficacy in the treatment of COVID-19. Numerous researchers and clinicians hypothesised that ascorbic acid could help prevent SARS-CoV-2 infection by boosting immune response and reducing the severity of the viral-mediated inflammatory response. Numerous studies support the conclusion that a high dose of the vitamin aids in immune system boosting [2].Numerous studies have been conducted to determine the effect of a high vitamin C consumption on the death rate of COVID-19 patients. While some studies have found no correlation between vitamin intake and mortality, others have discovered that this vitamin is beneficial in lowering the death rate [7].Vitamin C supplementation, according to a recent meta-analysis, reduces the length of stay in the intensive care unit by 8% and the duration of mechanical ventilation in ICU patients [38]. Another clinical trial conducted in three Chinese hospitals found that injecting large doses of vitamin C (50 mm every 12 hours for seven days) increased the pao2/fio2 ratio but failed to maintain it for days, and that increasing forceful mechanical ventilation for 28 days increased the pao2/fio2 ratio [6].

The duration of vitamin C treatment is also critical; some studies indicate that vitamin C is more effective when administered for at least seven days, and meta-analyses indicate that the ideal duration of vitamin C treatment for COVID-19 is seven days. When a disease lasts more than 3 to 4 days and the medication is used for less than 3 days, the efficacy decreases [7].

## 2. Gerneal background on vitmain C

The vitamin C found in fresh vegetables and fruit is vital to primates and pigs, for example (oranges, lemons, strawberries, broccoli, red peppers, and mangoes). Vitamin C serum and leukocyte levels drop during an infection's acute phase, according to research [8],[9]. Reproductive virus infections are less severe and last less time in people who have enough vitamin C in their diet. As opposed to the mild and controversial effects of oral vitamin C supplementation, an intravenous administration of high doses of vitamin C may lead to higher plasma levels by bypassing the limits of intestinal transporters. Since vitamin C increases the immune response to the new COVID, it may be used to treat COVID-19 according to this study (SARS-CoV-2). It has been known for the last quarter century that very ill patients, such as those with sepsis or multiple organ failure, have extremely low vitamin C levels [1],[5]. These severely ill individuals need larger doses of vitamin C to get their blood levels back to normal [8],[9] 20–30 times the amount that the average person needs [10],[11]. In patients with severe coronavirus disease, there is also a clear link between hospitalization in the intensive care unit (ICU) and vitamin C insufficiency, as a result, vitamin C is thought to be effective in preventing and treating coronavirus disease. Researchers found that giving IV (intravenous) vitamin C to sepsis patients helped reduce inflammatory indicators, tissue damage, and organ failure progress in a 2014 research led by fowler and his colleagues [12]. The quantity of vitamin C returned to low pre-infusion grades in some individuals, according to pharmacokinetic studies [8]. Sepsis and acute respiratory distress syndrome linked to COVID-19 may be helped by vitamin C components (ARDS). Current research indicates that SARS-CoV-2 infection is associated with depleted levels of the antiviral cytokine interferon [13] and that interferon levels are negatively correlated with disease severity [14],[15]. Aside from that, vitamin C boosts interferon levels in virally contaminated animals [16],[17]. Inflammatory markers are elevated in severe COVID-19, and this may sometimes cause a ‘cytokine storm’ [18].

Vitamin C also possesses antioxidant and anti-inflammatory effects that help to maintain equilibrium in this situation [19]. Evidence from the tiny COVID-19 exploratory study indicates that controlling the IV vitamin C dose may reduce IL-6 levels for up to seven days after the injection [20]. Chen and his colleagues examined the medical records of 29 people who had the COVID-19 virus. hs-CRP, a biomarker for oxidative stress and inflammation, was elevated in 27 of 29 patients (93 percent) [21]. Antioxidant response element (ARE) transcription factor nuclear factor erythroid-2-related factor 2 (nrf2) is a powerful regulator of cytoprotective protein expression-driven (ARE) anti-oxidant response element. Cells and tissues are protected against oxidative stress by activation of the nuclear factor erythroid 2–related factor 2 (Nrf2). In the body's anti-oxidant system, vitamin C plays a critical role [22],[23]. It also helps with severe care management. When infected with a virus or bacterium, a cytokine storm occurs [24], which leads to increased oxidative stress and, as a result, disease [25] This approach may be helpful for COVID-19 with a high intravenous vitamin C dosage based on the results of three previous clinical trials including 146 sepsis patients, since large quantities of anti-oxidant agents may recognise the counteraction and executives of oxidative stress [26]. Supplementing with high doses of oral Vitamin C may decrease the risk of viral infection and ameliorate symptoms [27],[28]. In order to treat 50 moderates to 20 g of vitamin C a day, given over an 8–10 h period of time, high-portion intravenous vitamin C is used. Patients in life-threatening situations need to take extra vitamin C tablets. In the end, all patients were healed and released from the hospital because of the better oxygenation record. A recent NIH expert board report says clearly that this regimen (1.5 g/kg body weight) is safe and free of significant regrettable occurrences [26]. In reality, large dosages of Vitamin C have been therapeutically used for quite some time. Experts in medical services should look at this possibility since it protects a significant quantity of vitamin C. There is a lot of clinical research planned, and the results are anticipated to establish particular bedside use standards. As a possible alternative to COVID-19 prevention and therapy, we shall examine the various pharmacological effects of vitamin C in this review. We'll also talk about whether or not taking large amounts of vitamin C is harmful.

## 3. Anti-viral characteristics of vitamin C

The antiviral action of vitamin C has been shown in many in vitro investigations, animal tests, and clinical trials. High dose of vitamin C has the potential to have a virucidal effect since it inhibits viral growth when multiplied in vitro. For the most part, vitamin C serves as a cofactor for enzymes like dioxygenases -ketoglutarate by neutralizing reactive oxygen species (ROS), rebuilding vitamin E, and reducing iron. Collagen cross-linking, gene expression control, and neurotransmitter production all require the usage of these enzymes. Chemotaxis and chemokinesis are only two ways ascorbic acid speeds up leukocyte activity, according to many studies [29] Vitamin C treatment in knockout mice (GULO) showed that the animals synthesis and produced interferon-alpha and beta against the influenza virus (H3N2), which cannot generate Vitamin C like humans [16]. Vitamin C has also been shown to be rich in the human brain, where it is believed to play important neuro-transmission functions, as well as neuro-protection and regulation [30] It has been studied that resting neutrophils contain high intracellular levels of vitamin C, around 1–2 mM, or about 10–100 fold higher than average plasma levels [31]. Vitamin C, an essential vitamin, has many roles in the body. The recommended daily consumption of vitamin C is 200 mg, which may be obtained through a healthy diet to keep your levels at their best. A 70 to 85 mol/L steady-state absorption occurs as a consequence [32]. Numerous clinical trials have shown that vitamin C has positive effects. Common winter illness symptoms, according to some, may be reduced by taking vitamin C regularly. It is possible to avoid URTIs by consuming 1–2 g of vitamin C each day. Studies have shown that consuming between 1 and 2 gram of vitamin C per day may help prevent illnesses of the upper respiratory tract (URTI) [33]. Maintaining a high level of vitamin C requires taking oral and inhalation supplements in combination. To prevent influenza virus' anti-viral impact by ensuring that respiratory secretions and bronchial epithelium have enough vitamin C [34]. Participation in 29 met-analysis studies shows that frequent ascorbic acid prophylaxis does not decrease common cold incidence in normal people by eleven thousand seven hundred and seven (11,707). However, six RTCs of ascorbic acid supplementation in 642, soldiers, skiers, and marathon runners showed a reduction in the frequency of the common cold [35].

In RCTs, participants face more physical stress, increasing their risk of infection compared to the general population [36]. Because of this, vitamin C (prophylactic) benefits have been seen in those at greater risk of getting the common cold [34]. RCTs have demonstrated that taking a daily supplement of 1 g of vitamin C may shorten the length of sickness, but this does not decrease the incidence of the common cold. The results of the meta-analysis indicate that taking an oral ascorbic acid supplement between 500 mg and 2 g/day has no effect on viral URTI incidence, but it does decrease the illness duration in children under the age of six years by 1.6 days [37]. More RCTs have shown that providing a daily dose of 30 mg zinc and 1 g of vitamin C may decrease common cold symptoms [37].According to a meta-analysis, adults who took over 0.2 gram of vitamin C per day, and children who took 1–2 g per day, had shorter and milder colds [37],[38].

**Figure 1.**
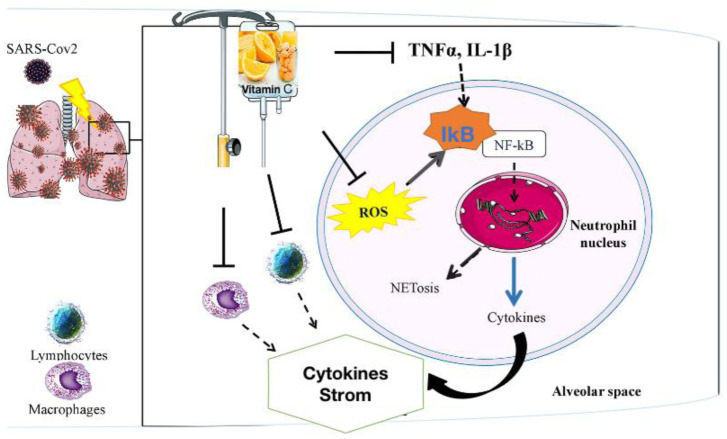
An IA administration of vitamin C to enhance the specific functions of immune system [11].

However, vitamin C treatment had no effect on the severity or duration of the common cold once symptoms started. In fact, even after the onset of symptoms, when vitamin C was administered at a dose of 3 g/day, the symptoms of the common cold improved [28]. Another study found that taking an extra-therapeutic dose of vitamin C combined with a regular daily intake of vitamin C as soon as symptoms emerged helped relieve cold symptoms and shorten the illness' duration [39]–[42]. Treatment with a mega-dose of vitamin C of 5–200 g/day may lessen AIDS symptoms and severity in infected individuals with Acquired Immunodeficiency Syndrome (AIDS) [40]. Vitamin C may be beneficial in the treatment of viral infections of the respiratory system and other viral diseases such as Herpes simplex virus (HSV-1), Vasilisa zoster and HIV, and its effectiveness is dose-dependent, according to the data from these clinical trials [43]. Free radicals are generated and phagocytes are activated as a result of most infections. ROS is tasked with the task of neutralizing viruses. Despite this, ROS are harmful to the host cell and may be involved in the pathogenesis. The respiratory syncytial virus causes infections of the upper and lower respiratory tract in infants and young children (RSV). Infected cells (epithelial) transmit RSV, resulting in the production of ROS that are resistant to pulmonary AO enzymes. RSV lung venomousness may be attributed in part to differences in cellular antioxidant-oxidant function [41].

## 4. Vitamin C and immune system

Ascorbic acid is a necessary nutrient for a wide range of immune functions. Ascorbic acid supplementation should have beneficial effects against various viral infections [41] Ascorbic acid, it is believed, accelerates immune function by increasing the censorious basal concentration of Vitamin C, which is required for the efficient and normal operation of the host's cover mechanism [42] An IA of vitamin C could influence specific functions of neutrophils (ROS and TNF, IL-1 mediated), blocking pathways involved in the formation of the Neutrophil Extracellular Trap (NETosis), and lowering uncontrollable inflammatory cytokine production in the alveolar region. In lymphocytes and macrophages, potential impacts on decreasing cytokine production have also been postulated. NFkB, nuclear transcription factor kappa B; inhibitory stimulus; dashed arrow, reduced effect or production (Figure 1).

It was discovered in an experiment that a low concentration of ascorbic acid may reduce cellular and humoral immune responses [44]. Ascorbic acid's effects on different populations of defense cells have also been explained in human and in vivo model experiments [45],[46]. Clinical research has shown that vitamin C medication promotes lymphocyte multiplication and natural killer cell chemotaxis in healthy subjects.

To top it all off, a high dosage of ascorbic acid non-individually revitalizes vermin immune cells, boosting production of interleukin-12 (IL) and the activity of B and T cells alike. Sepsis [47], ascorbate [48], sepsis-associated ascorbic acid Respiratory Distress Syndrome RDS [49], and more severe illnesses [9] have been identified in individuals infected by the virus. In order to destroy immune cells, ascorbic acids are required at all stages of the infection. When it comes to T-cell maturation, ascorbic acid is beneficial because it increases phagocytosis and apoptosis in exhausted neutrophils [50].Innate immune cells need to be kept active, functioning, differentiated, and proliferating [51]. Antimicrobial activities are increased, accompanying protein serum is promoted, and IFN g syntheses are stimulated [52],[53].Due to its ability to neutralise free radicals, ascorbic acid helps to keep the cellular redox balance in check (R-O). Achieving equilibrium within the short reaction time of the body's inherent resistance mechanism [35],[52],[53]. White (T) blood cells, particularly venom T cell receptor TCR cells, proliferate and differentiate in the plasma units, which helps produce antibodies via the proliferation and differentiation process [52].

It was shown in a meta-analysis that intravenous ascorbic acid treatment for ARDS and sepsis had benefits such as reduced vasopressor demands, a shorter stay in the intensive care unit (ICU), and less time in the hospital for censorious patients [54]. According to their findings, using more than 50 mg/day of ascorbic acid had a significant impact on the reduction of mortality rates in patients with sepsis-induced acute infection, according to Lin and colleagues. Excessive ascorbic acid treatment was shown to improve survival rates [25].

## 5. Vitamin C and its anti-oxidant effects

Reactive oxygen species (ROS) and attacks on their mobility are resisted by antioxidants or free-radical scavengers, resulting in reduced oxidative stress [55]. Using ascorbic acid as a treatment affects all of the proinflammatory cytokines being studied, and it may be the first choice [56]. Consumption of ascorbic acid or antioxidant supplements likely affects biochemical markers such as triglycerides, fetal bovine serum, and lipoprotein density [57] Large amounts of reactive oxygen species are the cause of damaged effects on many biological processes; these consequences are eliminated by the action of A.O (antioxidant) bacteria such as SOD, GSH-PX and CAT enzymes (catalase) e.g. As previously stated [58], all of these findings were attributed to Vitamin C's potent anti-oxidative action. An important antioxidant, ascorbic acid, is found in both the cell and plasma membranes of living organisms Scavengers function as antioxidants and increase the available Nitric Oxide (NO3) level via oxidative defense [59].

**Figure 2.**
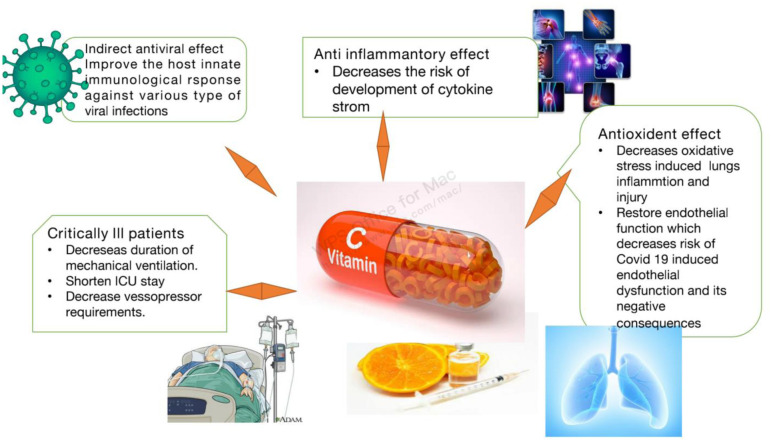
The possible beneficial effect of vitamin C in the management of COVID-19.

Moreover, vitamin C is historically and experimentally proved to ameliorate comorbid conditions in SARS-CoV-2 infected patients.

## 6. Role of vitamin C in the prevention of sepsis and pneumonia

When the alveoli in our lungs fill up with fluid, we have acute respiratory distress syndrome (ARDS), which is life-threatening. By holding on to the air, the fluid depleted our blood's supply of oxygen. Because of this, organs begin to fail, and as a result, the patient died. A large number of studies have shown that supplementing with vitamin C may help treat and even prevent respiratory infections. Many people have found success with vitamin C (C_6_H_8_O_6_) for respiratory infections including bronchitis and tonsillitis, as well as pneumonia and septic shock. Researchers found that intravenous [8] C_6_H_8_O_6_ 50 to 200 milligrammes each day for four days was given to sixteen individuals with Spartan sepsis who were sick from the disease [8]. Because to the use of ascorbic acid, the SOFA (sequential organ failure assessment) rate has been decreased the administration of 1 g of IV ascorbic acid every eight hours for 28 days to 594 critically sick patients showed a significant reduction in the incidence of multiorgan failure, the length of stay in ICU, and the duration of mechanical ventilation [60],[61].In earlier trials, researchers used intravenous vitamin C 1.5 g every 6 hours for four days, together with hydrocortisone and thiamine, on 47 ICU Septic patients. Patient's mortality rate and need for vasopressor decreased significantly after IV vitamin C administration, according to an analysis of all available data. Numerous follow-up tests on censoriously septic patients showed encouraging outcomes when ascorbic acid was used [49],[62]. However, other studies on ascorbic acid with ARDS septic patients came up empty [63],[64]. K. Al Sulaiman and colleagues use a regular and modest dosage of 1000 mg centrally once day in contrast to these studies [65].

## 7. Vitamin C and COVID-19

Viruses that cause COVID-19 illness have been found in Wuhan, China, on December 19, 2019. Breathing in infected aerosols increases its strong transmission capability, and after 3–14 days of incubation, it may be accountable for illness with symptomless to fatal effects [66]. Oxidative stress and a high tolerance for vitamin C corrosive high doses explain the disease's virulence. The anti-oxidant ascorbic acid is helpful in the presence of low levels of anti-oxidants because inflammation occurs when levels of anti-oxidants are low. Multiple studies have shown that using anti-oxidant supplements may help fight the virus COVID-19. Newly discovered beneficial outcomes after high doses of vitamin C therapy in clinical trials have elevated the nutrient's status as a leading therapeutic option for COVID-19. Due to its ability to enhance immunological function, the outcomes and patients are also very restricted. Ascorbic acid causes neutrophils to migrate to the site of infection and promotes phagocytosis in those cells.

Aside from that, ascorbic acid influences the differentiation and growth of both natural killer and T-cells [67],[68]. Previous studies revealed that the administration of vitamin C (125 and 250 mg/kg) to mice is connected with enhanced survival rates as well as a prolongation of the time surviavl. In addition, vitamin C has been shown to lower the levels of inflammatory cytokines in the blood four days following an infection. Aside from that, vitamin C has been shown to lower the levels of pro-inflammatory cytokines four days following an infection. They reached the conclusion that vitamin C could prevent influenza virus infection and subsequent pneumonia in a restraint-stressed mouse model by acting as an antiviral [68]. It has been demonstrated that vitamin C can lessen the likelihood of the formation of a cytokine storm during the late stage of COVID-19 in some patients [69],[70].Patients with moderate (10 g daily) and severe (20 g daily) COVID-19 may also benefit from high doses of intravenous vitamin C, which may help them improve their clinical outcomes. Furthermore, vitamin C supplementation has been shown to shorten the length of a hospital stay (3–5 days) [71],[72]. Aside from that, research have shown that combining vitamin C with other drugs can have positive benefits. Examples include the administration of vitamins C and E along with curcumin and glycyrrhizic acid, which have been demonstrated to reduce an excessive inflammatory response while also improving the innate antiviral immune response [73]. A further finding was that the administration of vitamin C in conjunction with diammonium glycyrrhizinate and quercetin resulted in a considerable reduction in the symptoms of non-hospitalized individuals with COVID-19 as well as a synergistic antiviral action [41].

**Figure 3.**
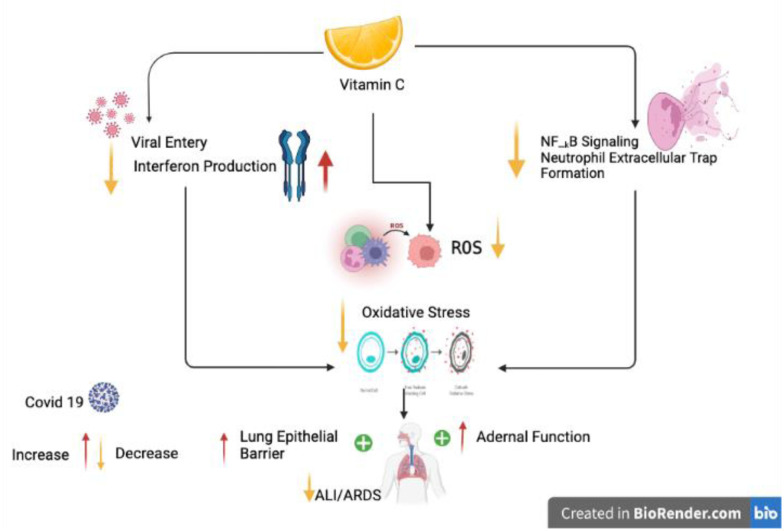
Postulated mechanisms for vitamin C's amelioration of COVID-19 pathology. *Note: ↓-decreased; ↑-increased; ALI-acute lung injury; ARDS-acute respiratory distress syndrome; NF-κB-nuclear factor kappa B.

Studies have shown a very low concentration of ascorbic acid in critically ill individuals [54]. According to a meta-analysis of eight studies, ascorbic acid may shorten the time patients are on mechanical breathing. A recent research involving seventeen individuals found that intravenous ascorbic acid helped treat infected persons with tolerated acute COVID-19 illness by reducing inflammatory marker levels. People with corona illness who are censoriously affected show improved behavior when given a higher dosage of ascorbic acid (24 g/day for 7 days) during trials performed in China (Wuhan) [74]. Ascorbic acid plasma levels in COVID-19 patients may now be measured using a new UPLC-MS or MS technique developed by new study. COVID-19 individuals tested positive for abnormally low levels of ascorbic acid, according to these findings. As a result, ascorbic acid is required as a COVID-19 infection supplement. More recent research shows that treating COVID-19-infected people with a higher dose of ascorbic acid may help their recovery [32]. Vitamin C is a safe and inexpensive essential nutrient and therefore investigation of its possible effects on COVID-19 should be encouraged along with the several other potential treatments. Currently, on Clinicaltrial.gov and the WHO's International Clinical Studies Registry Platform, many ongoing clinical trials are using Vitamin C alone or in combination with other medicines to examine the feasibility of treating COVID-19 illnesses (Table 1).

**Table 1.** Ongoing clinical trial for using Vitamin C for COVID-19 patient treatment.

S.No.	Study no	country	Participant	intervention	Register with
1	NCT04354428	USA	630 COVID-19 Patients	Trial arms: Vit C + folic acid Hydroxychloroquine +Folic Acid	Clinicaltrials.gov
2	NCT04264533	Wuhan, China	140 COVID-19 Patients	12 g Vitamin C, IV, every 12 hours	Clinicaltrail.gov
3	NCT04370288	Mashhad Iran	20 COVID-19 patients	MCN (Methylene blue, vitamin C, N-acetyl cysteine)	Clinicaltrial.gov
4	ChiCTR2000033050	Shanghai, China	110 COVID-19 patients	High dose of Vit C IV to COVID-19 patient	CHICTR.org.in
5	TCTR20200404004	Bangkok, Thailand	400 COVID-19 patients	Chloroquine 10 mg base/kg once in a 24 hour, Vit C 1000 mg in 24 hours.	Clinicaltrials.in.th
6	ChiCTR2000032717	Xian, Shaanxi, China	60 COVID-19 patients	High dose of vit C + Chinese medicine	CHICTR.org.in
7	NCT04357782	Virginia, USA	20 COVID-19 patients	Vit C (IV 50 mg/kg) 4 days, with an interval of 6 hours.	Clinicaltrial.gov
8	ACTRN12620000557932	Australia, Germany, USA	200 COVID-19 patients	1.Hydroxychloroquine + Zn+ Vit D3/B12+ azithromycin + IV Vit C 2.Hydroxychloroquine + zinc + Vit D3/B12 + azithromycin	WHO.ir
9	NCT04401150	Sherbrooke, Quebec, Canada	800 COVID-19 patients	Vit C (IV 50 mg/kg) every 6 hours	Clinicaltrial.gov
10	ChiCTR2000029768	Wuhan, China	600 COVID-19 patients	Diammonium Glycyrrhizinate Enteric-coated Capsules (oral, 150 mg, Tid), Oral Vit C tablets (5 g) every day	Chictr.org
11	NCT04323514	Palermo, Italy	500 COVID-19 patients	Vit C (IV 10 g) + conventional therapy	Clinicaltrial.gov
12	IRCT20200324046850N5	Abadan, Khuzestan Province, Iran	40 COVID-19 patients	Hydroxychloroquine 200 mg + Vit C (oral 500 mg) for 5 days with an interval of 12 hours	IRCT.ir
13	NCT04344184	Richmond, Virginia, USA	200 COVID-19 patients	Vit C (IV 100 mg/kg) every 8 hours	Clinicaltrial.gov
14	ChiCTR2000032716	Shanghai, China	12 COVID-19 patients	High dose of Vit C IV treatment upon diagnosis of severe COVID-19 patients	CHICTR.org.in
15	NCT04342728	USA	520 COVID-19 patients	1. 2–3 doses of Vit C 8000 mg 2. Zn Gluconate 50 mg daily 3.Vit C 8000mg + Zinc gluconate 50mg daily	Clinicaltrials.gov
16	NCT04395768	Victoria, Australia	200 COVID-19 patients	IV vitamin C, Hydroxycholorquine, azithromycin, Zinc citrate, Vitamin D3, Vitamin B12 for treating COVID-19	Clinicaltrials.gov
17	NCT03680274	Quebec, Canada	800 COVID-19 patients	Vit C (IV 50 mg/kg) every 6 hours for 96 hours	Clinicaltrial.gov
18	ChiCTR2000032400	Shanghai, China	120 COVID-19 patients	Vit C (IV 100 mg/kg) daily	WHO.int
19	NCT04363216	USA	66 COVID-19 patients	Increasing dose of Vit C oral (0.3 g/kg, 0.6 g/kg, 0.9 g/kg) every 6 hours	Clinicaltrial.gov

## 8. Conclusion

In summary, vitamin C possesses positive impacts on curing of infection and this may play a protective role in the current COVID-19 pandemic through boosting the immune system. Because of its antioxidant and anti-inflammatory properties, vitamin C is widely used in the treatment of a variety of diseases. Vitamin C functions as a powerful antioxidant, assisting in normal neutrophil function, scavenging ROS, regenerating vitamin E, modulating signaling pathways, activating pro-inflammatory transcription factors, activating the signaling cascade, nuclear factor B (NFB), regulating inflammatory mediators, gene regulation, phagocytosis, and signaling pathways in T cells, and increasing neutrophil motility to the site of infection. So, to develop strong immunity against COVID-19 infection, a regular administration of vitamin C is required.

Interesting results have been obtained by administering larger doses of IV Ascorbic acid to COVID-19 infected patients in China and the United States. Whether vitamin C may assist specifically in treating COVID-19 individuals who are elderly, have fundamental illnesses, or are members of African American populations, it would be interesting to see if this can be shown. Briefly summarised, high-dose vitamin C has been shown to reduce inflammation, improve oxygen support status, and reduce mortality in COVID-19 patients, all without causing any negative side effects. Additionally, it may be beneficial for specific subgroups of patients with severe and critical condition, as well as for older individuals. High-dose vitamin C may prove to be an effective treatment for COVID-19. Furthermore, there is an urgent need to investigate the direct relationship between serum/plasma nutritional C levels and the incidence and severity of COVID-19 infection.
